# Isomer Composition Assessment of Synthetic Phosphatidylethanol by Collision‐Induced and Ozone‐Induced Dissociation Mass Spectrometry

**DOI:** 10.1002/dta.70046

**Published:** 2026-02-17

**Authors:** Matthias Bantle, Jackson O. T. Long, Samuel C. Brydon, Reuben S. E. Young, Stephen J. Blanksby, Wolfgang Weinmann, Marc Luginbühl

**Affiliations:** ^1^ Institute of Forensic Medicine Bern, Forensic Toxicology and Chemistry University of Bern Bern Switzerland; ^2^ Department of Chemistry, Biochemistry and Pharmacy University of Bern Bern Switzerland; ^3^ Central Analytical Research Facility and School of Chemistry and Physics Queensland University of Technology Brisbane Queensland Australia; ^4^ Molecular Horizons and School of Science University of Wollongong Wollongong New South Wales Australia; ^5^ Institute for Clinical Chemistry University Hospital and University of Zurich Zurich Switzerland

**Keywords:** collision‐induced dissociation/ozone‐induced dissociation, glycerophospholipid, liquid chromatography–tandem mass spectrometry, PEth, phosphatidylethanol, regioisomers, *sn*‐positional isomers

## Abstract

The direct blood‐alcohol biomarker phosphatidylethanol (PEth), especially its most abundant analogue 1‐palmitoyl‐2‐oleoyl‐*sn*‐phosphatidylethanol (PEth 16:0/18:1) has gained increasing relevance in clinical and forensic applications for assessing alcohol consumption. Accurate quantification of PEth is essential to reliably differentiate between abstinence, moderate alcohol consumption and excessive alcohol intake. Measurement accuracy of PEth 16:0/18:1 by well‐established liquid chromatography–tandem mass spectrometry (LC‐MS/MS) approaches such as multiple reaction monitoring (MRM) can be confounded by the presence of the regioisomer 1‐oleoyl‐2‐palmitoyl‐*sn*‐phosphatidylethanol (PEth 18:1/16:0) in samples and synthetic reference standards. To address this measurement uncertainty, we conducted a new assessment of the isomeric composition of six currently available reference materials from four suppliers using collision‐induced dissociation/ozone‐induced dissociation (CID/OzID). Examination of these synthetic compounds found a high degree of regioisomeric purity of > 95%. Thus verified, the relative abundance of two key LC–MS/MS transitions were compared across a range of collision energies for both reference materials and an exemplary set of 10 dried blood spot case samples. These findings suggest a significantly wider range of natural isomer distributions spanning both higher and lower regiochemical composition (88.8%–98.85%) than the reference materials but within a range that would not significantly impact clinical classification.

## Graphical abstract

This study examines the isomeric composition of PEth reference materials and compares it with the natural variation in isomer distribution found in blood samples.

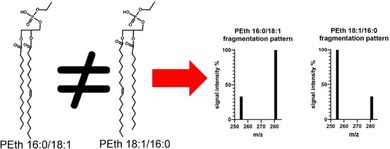

## 1 Introduction

Phosphatidylethanols (PEth) are a group of abnormal glycerophospholipids, each comprising a fatty acid at the *sn*‐1 and *sn*‐2 positions and a phosphoethanol head group at the *sn‐*3 position of a glycerol backbone [1]. As PEth is exclusively formed when ethanol is present in the body, levels in the blood are now used widely as a clinical marker for alcohol consumption [2, 3]. With a half‐life of 8–13 days, PEth can be detected in the blood for up to a month, depending on individual drinking patterns [4, 5]. PEth is used in clinical and forensic practice, including driving aptitude assessment and the detection and diagnosis of alcohol misuse or use disorders [6]. To date, at least 48 fatty acid combinations have been identified within the PEth subclass, with palmitoyl‐oleoyl‐phosphatidylethanol (PEth 16:0/18:1) being the most abundant species in human erythrocytes mirroring the similarly high abundance of its precursor palmitoyl‐oleoyl‐phosphatidylcholine (PC 16:0/18:1) [7, 8].

PEth is typically analysed using liquid chromatography–tandem mass spectrometry (LC–MS/MS) in negative ionization mode, employing multiple‐reaction monitoring (MRM) transitions based on the fatty acyl product anions formed upon collision‐induced dissociation (CID). As observed for other classes of glycerophospholipids, the strength of these transitions depends both on the identity of the fatty acyl chains but also on their relative (*sn*‐) position on the glycerol backbone [8, 9]. Previous LC–MS/MS investigations of PEth in MRM mode have demonstrated the preferential formation of the fatty acyl product ion from the *sn*‐2 position (central position) on the glycerol backbone and the sensitivity of the relative product ion abundance to collision energy (CE) [10, 11]. These findings emphasize the critical importance of matching both the acyl chain composition and the *sn*‐positional substitution of reference materials with the biomarkers themselves. In a previous study, Luginbühl et al. demonstrated that commercially available, synthetic PEth standards for palmitoyl‐oleoyl‐phosphatidylethanol were most often a mixture of 1‐palmitoyl‐2‐oleoyl‐*sn*‐phosphatidylethanol (PEth 16:0/18:1) and its regioisomer 1‐oleoyl‐2‐palmitoyl‐*sn*‐phosphatidylethanol (PEth 18:1/16:0) and noted that deviations in this regioisomeric composition from the naturally occurring composition could lead to significant quantification discrepancies [10, 12]. In response to these findings, several manufacturers have developed PEth 16:0/18:1 reference materials that claim high regioisomeric purity.

In this study, we investigated the *sn*‐positional isomer composition of newly introduced and commercially available PEth 16:0/18:1 reference materials. At the time of the study, the widely used PEth 16:0/18:1 reference material from Cerilliant was not available for purchase. Commercially available were three reference materials from one manufacturer (Avanti Polar Lipids) and additional materials from three other manufacturers (Chiron, Echelon Biosciences, Enzo Life Sciences), all of which claimed high purity. Additionally, some remaining material from an older Cerilliant batch was included for comparison. The proportion of non‐canonical PEth 18:1/16:0 in the synthetic PEth 16:0/18:1 materials was established using CID/ozone‐induced dissociation (OzID) technology that has previously been benchmarked for regioisomer quantification in glycerophospholipids [13, 14, 15]. Once benchmarked, the reference materials were assessed by LC–MS/MS in MRM mode across a wide range of CE, and a calibration curve was deployed to explore the natural isomer abundance variation across a set of 10 dried blood spot (DBS) case samples.

## 2 Materials and Methods

### 2.1 Chemicals and Reagents

PEth 16:0/18:1 (1‐palmitoyl‐2‐oleoyl‐*sn*‐glycero‐3‐phosphoethanol, powder, sodium salt, 840514P‐25 mg, MLOT: 5552PJF016, later referred to as ‘Avanti Mix’), PEth 16:0/18:1 IsoPure (1‐palmitoyl‐2‐oleoyl‐*sn*‐glycero‐3‐phosphoethanol, 10 mg/mL in chloroform, sodium salt, 792574C‐10 mg, MLOT: 6880CIB010) and PEth 18:1/16:0 IsoPure (1‐oleoyl‐2‐palmitoyl‐*sn*‐glycero‐3‐phosphoethanol, 10 mg/mL in chloroform, sodium salt, 792575C‐10 mg, MLOT: 6879CIA010) were purchased from Avanti Polar Lipids Inc. (Alabama, USA). PEth 16:0/18:1 (solid, ammonium salt, L‐6019‐5 mg, Lot No.: E00267‐121‐06) was obtained from Echelon Biosciences (Lubio Science, Zurich, Switzerland). PEth 16:0/18:1 (1 mg/mL in isopropanol, ammonium salt, C15121.39‐K‐IP, Lot No.: 32059) was purchased from Chiron Analytical Standards (Trondheim, Norway). PEth 16:0/18:1 (solid, BML‐ST400‐0010, Lot No.: 43MV33) was obtained from Enzo (Enzo Life Sciences AG, Lausen, Switzerland). PEth 16:0/18:1 (1 mg/mL in methanol, ammonium salt, P‐114, Lot No.: FN10222003) was purchased from Cerilliant (Round Rock, Texas, USA). Three external quality control (QC) samples (49WHA040‐005, Lot No.: 4170523166; 49WHS300‐005, Lot No.: 4170523167; 49WHA323, Lot No.: 8150323113) were obtained from ACQ Science (Rottenburg a.N., Germany) as lyophilisate. All solvents were of HPLC quality. Chloroform (CHCl_3_, 99%, p.a.) was obtained from Merck (Darmstadt, Germany). 2‐Propanol (≥ 99.5%) was purchased from Fisher Scientific (Loughborough, UK). Methanol (≥ 99.9%) was obtained from Biosolve (Valkenswaard, NL). Ultrahigh‐purity oxygen (≥ 99.999%) was purchased from Coregas (Sydney, Australia). Pentadeuterated PEth 16:0/18:1 (PEth 16:0/18:1‐D_5_) (L‐6051, Lot No.: E00298‐75‐09) was obtained from Echelon Biosciences (Salt Lake City, UT, USA).

### 2.2 LC–MS/MS in MRM Mode

The solid substances were each dissolved in 1‐mL CHCl_3_. Dilution series were performed in 2‐propanol yielding a concentration of 50 ng/mL for all substances. The solutions were analysed on an LC–MS/MS system consisting of an UltiMate 3000 HPLC system (Dionex, Thermo Scientific Instruments, Reinach, Switzerland) coupled to a 5500 QTRAP with TurboIonSpray source (Sciex, Toronto, Canada) operated in negative ionization mode with an ionization voltage of −4500 V. The two most intense MRM transitions (*m/z* 701.3/255.3 and 701.3/281.2) were used and ce were varied between −5 and −70 V in steps of 5 V with dwell times of 5 ms per transition. For all MRM transitions with *m/z* 701.3/255.3 and 701.3/281.2, declustering potential (DP) was −32 and −20 V, respectively. For all transitions, entrance potential (EP) was −10 V, and cell exit potential (CXP) was set to −14 V. A chromatographic run of 5.5‐min duration was employed using a Kinetex C18 2.6 μm 100 Å 50 × 2.1 mm (Phenomenex, Torrance, CA, USA) at an oven temperature of 50°C and a flow rate of 0.46 mL/min. Gradient elution was executed using MeCN/H_2_O 3:7 (v/v) with 5‐mM ammonium formate (Mobile Phase A) and MeOH/iPrOH/MeCN 1:1:1 (v/v) (Mobile Phase B): 0–1 min: 60% B, 1–2.6 min: 60%–90% B (linear), 2.6–3.2 min: 90%–100% B (linear), 3.2–4 min: 100% B, 4–4.1 min: 100%–60% B (linear), 4.1–5.5 min: 60% B. All substances were analysed in technical triplicate.

Ten case samples were selected, covering a concentration range of 20–270 ng/mL, and were reanalysed using the method described above. DBS samples were collected on DBS cards (STERA, Basel, Switzerland). Upon arrival of the samples in the laboratory, they were stored at −20°C to prevent PEth degradation. Additionally, one calibration and one QC sample from Cerilliant spiked into PEth‐negative blood from a teetotaler and three external QC samples from ACQ Science (Rottenburg a.N., Germany) were analysed. These calibration, internal and external QC samples are part of the currently used standards and QC samples for quantitative PEth analysis. All samples and standards were extracted from DBSs using methanol mixed with deuterated internal standard (PEth 16:0/18:1‐D_5_) (400 μL) in 96‐deep‐well plates (2‐mL square, Biotage, Uppsala, Sweden). Thereby, the DBS were cut out and placed in a 96‐deep‐well plate. The extraction solvent was then added, and after incubation at room temperature for 30 min, the supernatant was transferred into another 96‐deep‐well plate prior to analysis using the LC–MS/MS method described above. From the IsoPure standards from Avanti, three mixtures containing about 25/75, 50/50 and 75/25 (%v/v) PEth 16:0/18:1 and PEth 18:1/16:0, respectively, were prepared and analysed as described above generating a calibration for regioisomeric composition.

### 2.3 CID/OzID Mass Spectrometry

Avanti IsoPure PEth 16:0/18:1, Avanti IsoPure PEth 18:1/16:0 and ‘Avanti Mix‘ were prepared at 100 μM in methanol and were mixed 3:1 (v/v) with 500‐μM methanolic sodium acetate. All other PEth standards and mixtures (Avanti 25% 16:0/18:1, Avanti 50% 16:0/18:1 and Avanti 75% 16:0/18:1, Chiron, Echelon, Enzo) were prepared at 100 μM in methanol and were mixed 1:1 (v/v) with 500‐μM methanolic sodium acetate.

Analysis of the regioisomeric purity of the PEth standards and mixtures was performed using a modified hybrid linear ion‐trap Orbitrap mass spectrometer (LTQ Orbitrap Elite, Thermo Scientific, San Jose, CA, USA) as previously described [16]. Ozone was produced by passing ultrahigh‐purity oxygen through an ozone generator (Titan UHC, Absolute Ozone, Edmonton, Canada) before mixing with the helium buffer gas flowing into the high‐pressure region of the linear ion trap. CID/OzID analysis was undertaken using a procedure similar to that described by Luginbühl et al. [10] Briefly, samples were introduced using a chip‐based nano‐electrospray ionization (nESI) source (TriVersa Nanomate, Advion Interchim Scientific, Ithaca, NY, USA), operating in positive ionization mode, delivering 15‐μL injections of sample from a 96‐well plate. Using the instrument software (Xcalibur 4.7, Thermo Scientific), parameters for analysis at the MS^2^ and MS^3^ levels were set for high‐resolution MS analysis of the CID/OzID product ions as follows. The sodiated PEth precursor ion ([M + Na]^+^, *m/z* 725.5) was mass‐selected with an isolation width of 3 Da and subjected to CID using a normalized CE (NCE) of 30 and an activation time of 10 ms. The CID product ion at *m/z* 599.5 was reisolated using an isolation width of 3 Da and trapped in the presence of ozone for 500 ms without NCE. Diagnostic CID/OzID product ions at *m/z* 275, 291, 319, 345, 379, 395, 405 and 421 were extracted with ion abundances normalized to the total ion current and averaged over 100 individual scans (error ±1*σ*). The contribution of PEth 16:0/18:1 was calculated as a sum of the abundance of the diagnostic ions for PEth 16:0/18:1 (*m/z* 275, 291, 319, 379 and 395) over the abundance of the combined diagnostic ions for both isomers (*m/z* 275, 291, 319, 345, 379, 395, 405 and 421). The reported uncertainty was determined by propagation of error over at least four technical replicates for the Avanti standards and mixes used for the calibration curve and two replicates for the other commercial suppliers.

## 3 Results and Discussion

### 3.1 Regiochemical Composition Assessment of PEth References by CID/OzID

**FIGURE 1:**
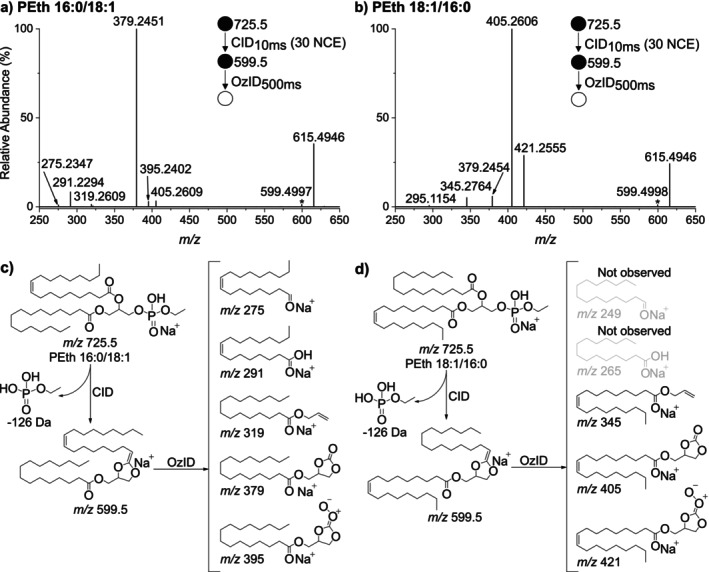
CID/OzID MS3 mass spectra obtained by trapping the CID product *m/z* 599.5 ions (*) in the presence of ozone for 500 ms. The *m/z* 599.5 ions were formed from CID of the [M + Na]^+^ ions (*m/z* 725.5) from synthetic standards (a) PEth 16:0/18:1 and (b) PEth 18:1/16:0. CID/OzID fragmentation schemes for (c) PEth 16:0/18:1n‐9,cis and (d) PEth 18:1n‐9,cis/16:0.

As described previously for PEth [10], and for other glycerophospholipid subclasses [13, 14, 15], CID of the sodiated PEth 16:0/18:1 molecular ion (*m/z* 725.5) promotes loss of the phosphate head group via a cyclic‐acetal rearrangement of the glycerol backbone. This results in the formation of a new carbon–carbon double bond at the acetal carbon of the acyl chain bound at the *sn‐*2 position. Reisolation and trapping of this product ion (*m/z* 599.5) in the presence of ozone causes fragmentation of the double bond to yield characteristic ions indicative of the remaining fatty acyl chain at the *sn*‐1 position with the accompanying neutral loss of the *sn*‐2 fatty acyl chain substituent. Here, CID/OzID was initially applied to the IsoPure PEth 16:0/18:1 and PEth 18:1/16:0 standards supplied by Avanti (Figure 1a,b). After 500 ms in the presence of ozone, the MS^3^ spectra show the diagnostic ions for each *sn*‐regioisomer with the base peaks being *m/z* 379 for PEth 16:0/18:1 and *m/z* 405 for PEth 18:1/16:0, in line with the mechanisms outlined in Figure 1c,d, respectively. In addition, a series of less abundant product ions were observed that could be associated exclusively with each isomer including *m/z* 395, 319, 291 and 275 arising from the canonical PEth 16:0/18:1 isomer (Figure 1c) while *m/z* 421 and 345 could be attributed to the non‐canonical PEth 18:1/16:0 isomer (Figure 1d). Notably, the spectra obtained for each isomer also showed marker ions for the alternative *sn*‐regioisomer, indicative of up to 5% of the alternate regioisomer.

CID/OzID product ion abundances have previously been calibrated against phospholipase A2 (PLA_2_) enzyme assays demonstrating that the former can be treated quantitatively for assessing the regiochemical composition of glycerophospholipids [13, 14]. The composition of the reference materials as well as the artificial mixtures prepared from Avanti PEth 16:0/18:1 and PEth 18:1/16:0 (25/75, 50/50 and 75/25 [%v/v]) are compared with their manufacturer‐reported composition or mixture stoichiometry in Table 1. All of the reference materials with claimed high purity (Avanti IsoPure PEth 16:0/18:1, Chiron, Echelon and Enzo) showed a PEth 16:0/18:1 composition of > 96.1% with errors < 1.5%, consistent with the regioisomeric composition reported by the manufacturers. Furthermore, the Avanti IsoPure of the non‐canonical PEth 18:1/16:0 also showed minimal *sn*‐regioisomeric impurity with only 4.7% ± 1.3% PEth 16:0/18:1 present. The resulting binary mixtures of the two Avanti IsoPure references had minimal deviations from the calculated nominal ranges of regioisomeric composition (Table 1). Noting that additional pipetting imprecisions upon sample preparation could not be excluded. The commercially available PEth 16:0/18:1 from Avanti that is not regioisomerically pure (Avanti Mix) was found to be compositionally 80.3% ± 1.7% PEth 16:0/18:1 and is consistent with the 75.8% ± 1.5% determined for an older batch of the same product [10].

**TABLE 1:** Comparison of reported or nominal composition and composition determined by CID/OzID for the reference standards and the three mixtures (25%, 50% and 75%) prepared from Avanti IsoPure PEth 16:0/18:1 and PEth 18:1/16:0.

Material	Reported or nominal % [16:0/18:1]/[16:0/18:1 + 18:1/16:0]	CID/OzID % [16:0/18:1]/[16:0/18:1 + 18:1/16:0]	Error (%)
Avanti IsoPure 16:0/18:1	> 95%^a^	97.6	1.4
Avanti IsoPure 18:1/16:0	< 5%^a^	4.7	1.3
Avanti 25% 16:0/18:1	23.8–28.8^b^	25.6	0.8
Avanti 50% 16:0/18:1	47.5–52.5^b^	46.5	1.4
Avanti 75% 16:0/18:1	71.3–76.3^b^	72.4	0.8
Avanti Mix	Not stated^c^	80.3	1.7
Chiron	> 95%	96.1	0.4
Echelon	Not stated^c^	96.9	0.4
Enzo	Not stated^c^	96.5	0.7
Cerilliant^d^		99.2	1.5

^a^ Manufacturer states < 5% acyl chain migration by ^13^C‐NMR.

^b^ Calculated range based on < 5% acyl migration in each IsoPure.

^c^ Manufacturer does not state regioisomeric purity.

^d^ Cerilliant data were included from a previous study with Lot No.: FN10161801 [10].

### 3.2 LC–MS/MS in MRM Mode—Calibration and Analysis of PEth in Case Samples

**FIGURE 2:**
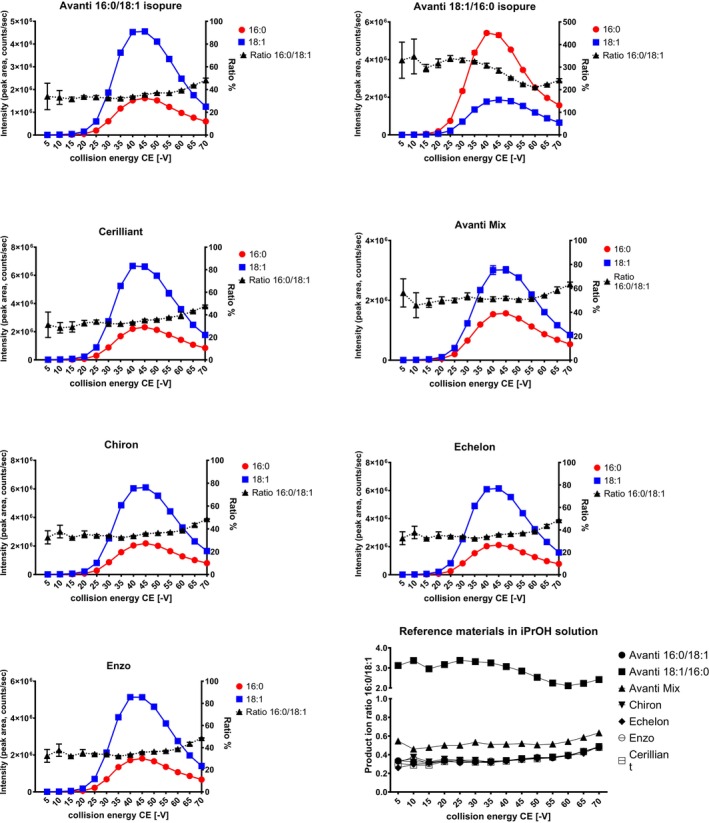
Absolute intensities and product ion ratios (16:0–18:1) for the different PEth 16:0/18:1 reference materials for collision energies ranging from −5 to −70 V. For visibility reasons, error bars are not shown in the bottom right figure.

With the regioisomeric composition of the various standards and mixtures established by CID/OzID, the use of appropriate CID conditions for LC–MS/MS in MRM mode could be investigated. For all pure substances, except PEth 18:1/16:0 from Avanti, the absolute intensity of the MRM 701 ⟶ 281 transition (later referred to as 18:1) was larger than the MRM 701 ⟶ 255 transition (later referred to as 16:0). With CE ranging from −5 to −70 V, the maximum intensity for both transitions was observed at a CE of −40 V and provided sufficient signal for the product ions compared with lower CEs (< −20 V). The mean ion ratio (16:0–18:1) at CE = −40 V for the claimed regioisomerically pure substances (Avanti PEth 16:0/18:1 IsoPure and the products from Chiron, Echelon and Enzo) and the previously established regioisomerically pure product from Cerilliant [10] was 0.335 ± 0.003. The product ion ratio for the ‘Avanti Mix’ (not regioisomerically pure) was 0.511 ± 0.015, whereas the ratio for Avanti PEth 18:1/16:0 IsoPure was 3.068 ± 0.084 (Figure 2). There was an overall trend to increasing ratios for higher CE.

**FIGURE 3:**
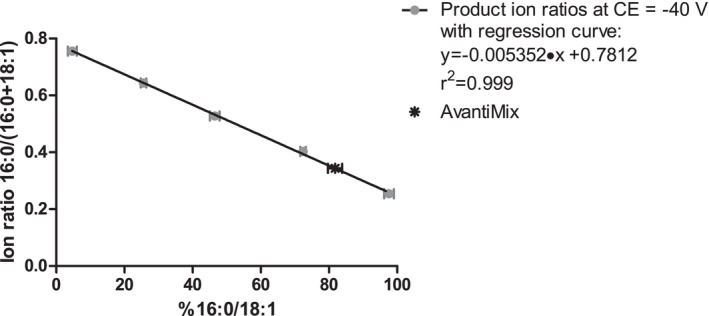
Calibration for regioisomeric composition at a collision energy of −40 V (full circles) with a linear regression (black line) with = −0.005352*x* + 0.7812 (*R* ^2^ > 0.99). The asterisk marks the product ion ratio (0.343) and the modelled PEth 16:0/18:1 content (82.78%) for ‘Avanti Mix’.

**FIGURE 4:**
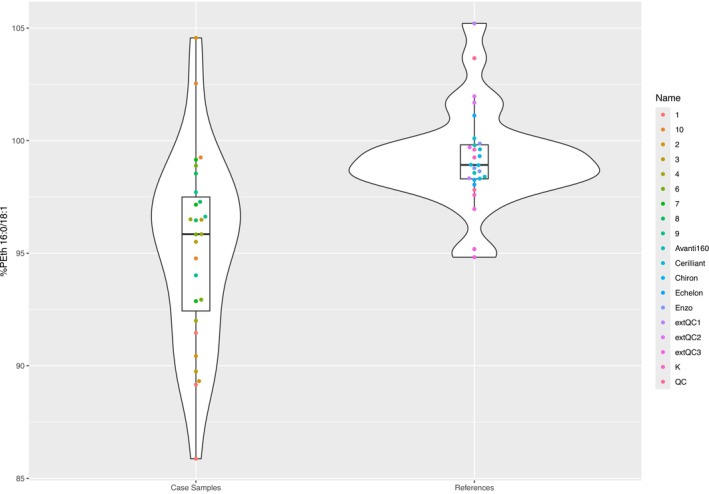
Violin plot of the comparison of modelled %PEth 16:0/18:1 composition for case samples (measured in triplicate, 27 datapoints used) and reference solutions (Avanti IsoPure 16:0/18:1, Echelon, Enzo, Cerilliant standards, Cerilliant calibration and QC in matrix and ACQ QCs in matrix [measured in triplicate, 28 datapoints used]).

For the calibration using three mixtures of the Avanti IsoPure PEth 16:0/18:1 and Avanti IsoPure PEth 18:1/16:0 compounds (nominal composition of 25%, 50% and 75% PEth 16:0/18:1) and the compounds themselves, a linear regression with y=−0.005352x+0.7812 was determined (*R* ^2^ > 0.99) at CE = −40 V with *x* being the content of PEth 16:0/18:1 in % determined by CID/OzID (Table 1) and *y* being the product ion fraction of 16:0/(16:0 + 18:1) from the LC–MS/MS experiments in MRM mode (Figure 3). Based on the linear regression, a PEth 16:0/18:1 content of 82.78% ± 1.26% was calculated for the ‘Avanti Mix’ product (product ion fraction of 0.338 ± 0.0067). For the other reference materials, contents of 98.8%–99.6% were calculated based on the regression curve from Figure 3; see Figure 4 and Table 2.

**TABLE 2:** Summary of the data underlying Figure 4.

Material type	Name	Fraction Rep1	Fraction Rep2	Fraction Rep3	%PEth 16:0/18:1 Rep1	%PEth 16:0/18:1 Rep2	%PEth 16:0/18:1 Rep3	Mean fraction	Mean %PEth 16:0/18:1
Reference	Avanti 160	0.255	0.247	0.254	98.40	99.79	98.56	0.252	98.92
Reference	Avanti 181	0.755	0.760	0.748	4.97	3.94	6.28	0.754	5.06
Reference	Avanti Mix	0.329	0.345	0.340	84.47	81.45	82.42	0.338	82.78
Reference	Chiron	0.252	0.250	0.255	98.91	99.31	98.31	0.252	98.84
Reference	Echelon	0.255	0.240	0.256	98.26	101.11	98.04	0.251	99.14
Reference	Enzo	0.253	0.253	0.247	98.78	98.64	99.87	0.251	99.10
Reference	Cerilliant	0.248	0.252	0.245	99.61	98.93	100.10	0.248	99.55
Cal/QC in matrix	QC	0.210^a^	0.258	0.226	106.66^a^	97.81	103.66	0.231	102.71
Cal/QC in matrix	K	0.259	0.250	0.248	97.59	99.25	99.60	0.252	98.81
extQC in matrix	1	0.218	0.310^a^	0.255	105.21	87.98^a^	98.33	0.261	97.17
extQC in matrix	2	0.248	0.237	0.235	99.70	101.69	101.97	0.240	101.12
extQC in matrix	3	0.262	0.274	0.272	96.96	94.82	95.18	0.269	95.66
Case sample	1	0.322	0.304	0.292	85.87	89.16	91.46	0.306	88.83
Case sample	2	0.222	0.303	0.297	104.56	89.32	90.43	0.274	94.77
Case sample	3	0.270	0.265	0.301	95.51	96.48	89.75	0.279	93.91
Case sample	4	0.265	0.289	0.268	96.50	92.00	95.85	0.274	94.78
Case sample	5	0.159^a^	0.264^a^	0.193^a^	116.17^a^	96.60^a^	109.98^a^	0.205^a^	107.58^a^
Case sample	6	0.268	0.284	0.252	95.83	92.93	98.89	0.268	95.89
Case sample	7	0.284	0.251	0.261	92.87	99.15	97.15	0.265	96.39
Case sample	8	0.265	0.261	0.254	96.46	97.28	98.54	0.260	97.43
Case sample	9	0.264	0.278	0.258	96.62	94.02	97.71	0.267	96.12
Case sample	10	0.250	0.232	0.274	99.25	102.54	94.77	0.252	98.85

^a^ Outliers, according to Grubbs' test, *α* = 5%.

For the calibration and QC samples from Cerilliant and the QC samples from ACQ, an average product ion fraction of 0.248 ± 0.006 (measured in triplicate, 13 datapoints in total, after removal of two outliers according to Grubbs' test, *α* = 5%) was found. The PEth 16:0/18:1 composition of all reference solutions with acclaimed high regioisomeric purity was 99.1% ± 2.1% (Figure 4 and Table 2), slightly higher than that determined by CID/OzID (Table 1). The calibration and QC samples from Cerilliant in matrix and the QC samples from ACQ Science were determined to have a similarly high composition of PEth 16:0/18:1 of 99.4% ± 2.7%. The high degree of regioisomeric purity determined for the nowadays available reference materials is in contrast to earlier findings from Luginbühl et al. They found varying product ion ratios (16:0/18:1) for different manufacturers at a CE of −30 V (slightly lower than the CE of −40 V used here), ranging from 0.31 to 0.45 and hence regioisomeric purities of 75.8%–99.2% [10]. However, the absolute values for product ion ratios are comparable and suggest that more manufacturers are producing *sn*‐regiopure reference materials compared with 5 years ago.

For the 10 case samples that were measured in triplicate, an average product ion fraction of 0.272 ± 0.01 (measured in triplicate, 27 measurements, in total after removal of three outliers according to Grubbs' test, *α* = 5%, range 0.253–0.306) was found. Using the calibration curve described above, a PEth 16:0/18:1 composition of 95.2% ± 3.2% (27 datapoints, range 88.8%–101.8%) was determined for the case samples (Figure 4). The reference materials were exhibiting a higher PEth 16:0/18:1 composition than the average of the case samples. In human blood samples, Ekroos et al. found a regioisomeric composition of 85% PC 16:0/18:1 [8], the biological precursor to PEth 16:0/18:1 as mentioned. However, care must be taken for in‐depth interpretation, as those values were at the limit or exceeding the range of the calibration. Furthermore, the small sample size limits the generalizability of these findings.

## 4 Conclusion

Using both CID/OzID and LC–MS/MS in MRM mode, regioisomeric composition has been determined for PEth 16:0/18:1 reference solutions from different manufacturers. The IsoPure PEth 16:0/18:1 from Avanti and the products from Cerilliant, Chiron, Echelon and Enzo exhibited a high degree of regioisomeric purity with more than 96% PEth 16:0/18:1 content. It appears that reference material manufacturers have recognized the importance of regioisomeric purity and have since provided various suitable materials. Thereby, the product ion ratios obtained from the references were in close agreement with the ratios found for exemplary case samples. Batch‐to‐batch variations cannot be fully excluded and therefore, regioisomeric purity should be tested or compared with solutions with known composition. The set‐up of a calibration and analysis by CID using material with known composition to determine the regioisomeric purity of a material in question is an alternative to CID/OzID, which requires ozonolysis capabilities. However, for the method involving CID, knowledge of the precise composition of the known reference materials is required, while CID/OzID is an absolute method. In comparison to the regioisomerically pure reference materials, the case samples were exhibiting a wider range of regioisomeric composition (88.8%–98.85%). Regarding the impact of regioisomeric purity on reliable quantification, we refer to a previous publication by Luginbühl et al. on this topic [10]. Depending on the selected MRM transition for PEth quantification, both signal enhancement and signal attenuation may occur. Further investigations, including regioisomeric analysis of both PC and PEth 16:0/18:1 in a larger cohort of case samples, could offer deeper insights into natural variability.

## Funding

This study was supported by the German Society for Traffic Medicine (DGVM) through the awarding of the Scientific Young Investigator Award to Marc Luginbühl. Jackson O. T. Long acknowledges his postgraduate scholarship sponsored by QUT and Adepa Lifesciences. Stephen J. Blanksby thanks the Australian Research Council (ARC) for the generous financial support through the Discovery Program (DP190101486).

## Ethics Statement

The use of anonymized biological material does not fall within the scope of the Swiss Human Research Act (Art. 2 HRA). All samples used in this project were originally collected for routine phosphatidylethanol determination. Any remaining material was subsequently anonymized, and the case samples used could no longer be attributed to any identifiable individual.

## Conflicts of Interest

The authors declare no conflicts of interest.

## Data Availability

The data that support the findings of this study are available from the corresponding author upon reasonable request.
